# Non-Pharmacological Interventions for Autism Spectrum Disorder in Greece: Evidence and Public Health Implications (2019–2022)

**DOI:** 10.1192/j.eurpsy.2026.11309

**Published:** 2026-07-31

**Authors:** R. Kouznetsov, I. Gounaridis, S. Moulinos, A. Konstantatou, P. Gourzis, I. Dimakos, E. Jelastopulu

**Affiliations:** 1 Department of Public Health, Epidemiology and Quality of Life, School of Medicine; 2 Department of Computer Engineering and Informatics, University of Patras, Patras; 3 Department of Digital Media and Communication, Ionian University, Corfu; 4 Medical School; 5 Department of Psychiatry, School of Medicine; 6 Department of Educational Sciences and Social Work, University of Patras, Patras, Greece

## Abstract

**Introduction:**

Autism spectrum disorder (ASD) is increasingly recognized as a major public health priority worldwide. Accurate prevalence estimates and systematic monitoring of therapeutic practices are essential for guiding healthcare planning, educational provision, and social support services. Although smaller-scale studies have previously explored ASD in Greece, no nationwide epidemiological study has examined non-pharmacological treatments prescribed to children aged 2–17 years.

**Objectives:**

This study presents the first comprehensive national analysis of non-pharmacological interventions between February 2019 and February 2022, providing a foundation for clinical services and policy action. Specifically, it aimed to quantify the frequency and distribution of therapies and to identify disparities in therapeutic practices that may inform future clinical, educational, and public health strategies.

**Methods:**

A retrospective analysis was conducted using anonymized administrative data from the Greek National Organization for Healthcare Services Provision (EOPYY). Variables included sex, age, ICD-10 diagnosis codes, and recorded treatment interventions. Statistical and geospatial analyses were performed in Python with pandas, matplotlib, and geopandas.

**Results:**

During the 3-year period, **18,502 children** aged 2–17 years were diagnosed with autism spectrum disorder (ASD) in Greece (14,678 boys and 3,824 girls). A total of **15,328,327 non-pharmacological therapies** were administered, 12,298,964 to boys and 3,029,363 to girls, corresponding to an overall average of **829 therapy sessions per child** (~276 per year). Per-child averages by therapy type were: speech therapy (233 for boys, 236 for girls), occupational therapy (219 vs. 255), special education (177 vs. 144), individual psychotherapy (162 vs. 122), behavioral therapy (43 vs. 32), family therapy (4 for both sexes), and fewer than one physiotherapy session for both.

**Conclusions:**

This study provides the first nationwide evidence on the scale and type of non-pharmacological interventions for children with ASD in Greece. The findings reveal a disproportionate reliance on speech therapy, occupational therapy, and special education, while family therapy remains almost absent as an intervention, despite international best practices demonstrating its crucial impact on child development and caregiver well-being. These imbalances reflect systemic gaps in service design, training, and policy prioritization. Addressing these shortcomings requires a paradigm shift: moving beyond a narrow focus on individual therapies to a more integrated model that includes families as active partners in care. Without this shift, the Greek health and education system risk perpetuating inequities in access, limiting therapeutic effectiveness, and neglecting the broader psychosocial needs of children with ASD and their families.

**Disclosure of Interest:**

None Declared

